# Time from Start of Quarantine to SARS-CoV-2 Positive Test Among Quarantined College and University Athletes — 17 States, June–October 2020

**DOI:** 10.15585/mmwr.mm7001a2

**Published:** 2021-01-08

**Authors:** Christine Atherstone, Meaghan L. Peterson, Mackenzie Malone, Margaret A. Honein, Adam MacNeil, Catherine S. O’Neal, Stephen Paul, Kimberly G. Harmon, Kyle Goerl, Cameron R. Wolfe, Julie Casani, Lisa C. Barrios, COVID-19 Collegiate Athlete Testing Group, COVID-19 Collegiate Athlete Testing Group, Douglas Aukerman, James E. Bray, Charlz Bisong, January Cornelius, Michaila Czarnik, Zarina Fershteyn, Metrecia Terrell, David Bui, Craig Kassinger, Dametreea Carr McCuin, Randy P. Cohen, Todd P. Daniels, Bruce Helming, David Millward, Donald Porter, James R. Clugston, Ron Courson, Anna Randa, Jon Divine, Calvin Duffaut, Katherine Fahy, Los Angeles, Nicolas Hatamiya, Amanda Honsvall, Emily Miller, Mark Pocinich, Los Angeles, Aurelia Nattiy, Annabelle de St. Maurice, Daniel Vigil, Michael D. Goodlett, Kent Hagan, Michele Kirk, James A. Madaleno, Jonathan Merkle, Kim Moon, Janna Sutherland, Timmy Pierce, Daniel Vinson, James B. Robinson, Takamasa Sakomato, Luis D. Salazar, Mollie Selfridge, Kevin Shubow, Owen Stanley, Stevan P. Whitt

**Affiliations:** ^1^Epidemic Intelligence Service, CDC; ^2^CDC COVID-19 Response Team; ^3^Louisiana State University Health Sciences Center, Baton Rouge, Louisiana; ^4^University of Arizona, Tucson, Arizona; ^5^University of Washington School of Medicine, Seattle, Washington; ^6^University of Kansas School of Medicine, Kansas City, Kansas; ^7^Duke University Medical Center, Durham, North Carolina; ^8^North Carolina State University, Raleigh, North Carolina.; Oregon State University, Corvallis, Oregon; University of Texas; Austin; 4ES Corporation, San Antonio, Texas; 4ES Corporation, San Antonio, Texas; 4ES Corporation, San Antonio, Texas; 4ES Corporation, San Antonio, Texas; 4ES Corporation, San Antonio, Texas; CDC; CDC; University of Arizona, Tucson, Arizona; University of Arizona, Tucson, Arizona; University of Arizona, Tucson, Arizona; University of Arizona, Tucson, Arizona; University of Arizona, Tucson, Arizona; University of Arizona, Tucson, Arizona; University of Florida, Gainesville, Florida; University of Georgia, Athens, Georgia; University of Georgia, Athens, Georgia; University of Cincinnati, Cincinnati, Ohio; University of California, Los Angeles; University of California; University of California, Los Angeles; University of California, Los Angeles; University of California, Los Angeles; University of California; University of California, Los Angeles; University of California, Los Angeles; University of California, Los Angeles; Auburn University, Auburn, Alabama; Advanced Orthopaedic Specialists, Fayetteville, Arkansas; Texas Christian University, Fort Worth, Texas; University of Kentucky, Lexington, Kentucky; University of Montevallo, Montevallo, Alabama; University of Montevallo, Montevallo; Alabama; University of Montevallo, Montevallo, Alabama; Oak Ridge Institute for Science and Education, Oak Ridge, Tennessee; Oak Ridge Institute for Science and Education, Oak Ridge, Tennessee; University of Alabama, Tuscaloosa, Alabama; University of Colorado, Boulder, Colorado; University of Kansas, Kansas City, Kansas; Stanford University, Stanford, California; University of California, Berkeley; University of Missouri, Columbia, Missouri; University of Missouri, Columbia, Missouri

To safely resume sports, college and university athletic programs and regional athletic conferences created plans to mitigate transmission of SARS-CoV-2, the virus that causes coronavirus disease 2019 (COVID-19). Mitigation measures included physical distancing, universal masking, and maximizing outdoor activity during training; routine testing; 10-day isolation of persons with COVID-19; and 14-day quarantine of athletes identified as close contacts* of persons with confirmed COVID-19. Regional athletic conferences created testing and quarantine policies based on National Collegiate Athletic Association (NCAA) guidance (*1*); testing policies varied by conference, school, and sport. To improve compliance with quarantine and reduce the personal and economic burden of quarantine adherence, the quarantine period has been reduced in several countries from 14 days to as few as 5 days with testing (*2*) or 10 days without testing (*3*). Data on quarantined athletes participating in NCAA sports were used to characterize COVID-19 exposures and assess the amount of time between quarantine start and first positive SARS-CoV-2 test result. Despite the potential risk for transmission from frequent, close contact associated with athletic activities (*4*), more athletes reported exposure to COVID-19 at social gatherings (40.7%) and from roommates (31.7%) than they did from exposures associated with athletic activities (12.7%). Among 1,830 quarantined athletes, 458 (25%) received positive reverse transcription–polymerase chain reaction (RT-PCR) test results during the 14-day quarantine, with a mean of 3.8 days from quarantine start (range = 0–14 days) until the positive test result. Among athletes who had not received a positive test result by quarantine day 5, the probability of having a positive test result decreased from 27% after day 5 to <5% after day 10. These findings support new guidance from CDC (*5*) in which different options are provided to shorten quarantine for persons such as collegiate athletes, especially if doing so will increase compliance, balancing the reduced duration of quarantine against a small but nonzero risk for postquarantine transmission. Improved adherence to mitigation measures (e.g., universal masking, physical distancing, and hand hygiene) at all times could further reduce exposures to SARS-CoV-2 and disruptions to athletic activities because of infections and quarantine (*1*,*6*).

CDC partnered with representatives of the NCAA conferences to analyze retrospective data collected by participating colleges and universities. A request for participation was sent to all NCAA members regardless of whether athletics programs had resumed. Colleges and universities were provided with a data dictionary to standardize data collection (Supplementary Table, https://stacks.cdc.gov/view/cdc/99431). Deidentified, individual-level retrospective testing and exposure data from quarantined, SARS-CoV-2–exposed collegiate athletes across the United States were provided to CDC for analysis. Information on the types of exposure was collected by athletic staff members or public health professionals and categorized as sports settings (e.g., game, practice, team workouts, scrimmage, shared equipment, or team travel), roommate, social gatherings (e.g., party, shared car, or friends), unspecified or other (e.g., class, meetings, work, or travel unrelated to sports). Data cleaning and analyses were conducted in duplicate using SAS (version 9.4; SAS Institute) and R (version 3.6.3; The R Foundation). This activity was reviewed by CDC and was conducted consistent with applicable federal law and CDC policy.^†^

For this analysis, athletes were excluded if they 1) had not received testing using RT-PCR during their 14-day quarantine period; 2) had received a positive SARS-CoV-2 RT-PCR test result before starting quarantine; 3) were still under quarantine and had not had positive test results at the time of data submission (October 27–November 17); or 4) were missing data on quarantine start date, test date, test type (i.e., antigen or RT-PCR), or RT-PCR test result (positive, negative, or indeterminate). For all athletes who received a positive SARS-CoV-2 RT-PCR test result during quarantine, time-to-event analyses were conducted by separately calculating the interval from the exposure date or the quarantine start date to the positive specimen collection date. Quarantined athlete data were excluded from time-to-event analyses if the exposure date or the quarantine start date was missing or if the athlete had positive test results >14 days after commencing quarantine. Kaplan-Meier survival analysis estimated the probability and 95% confidence intervals (CIs) of athletes receiving a first positive SARS-CoV-2 RT-PCR test result after days 5, 7, and 10 of quarantine.

Twenty-four colleges and universities contributed data on 2,257 quarantined athletes, irrespective of test result; data from 427 athletes were excluded based on the described exclusion criteria. Among the remaining 1,830 quarantined athletes, the most common sports played were football (46.2%, 846), track and field or cross-country (10.4%, 190), and soccer (6.6%, 121) (Table). The most commonly reported exposures were at social gatherings (40.7%, 745) or from roommates (31.7%, 580); overall, 232 (12.7%) quarantined athletes reported exposure associated with athletic activities. Athletes received a total of 3,345 RT-PCR tests (mean = 1.8 per athlete, range = 1–14) while in quarantine. A total of 458 (25.0%) quarantined athletes ever received a positive test result, including 137 (29.9%) who never reported symptoms. Among 386 quarantined athletes who became symptomatic, 321 (83.2%) ever received a positive test result.

**TABLE:** Sports played, symptoms, and exposure type among quarantined college and university athletes with COVID-19 exposure *—* 17 states, June*–*October 2020

Sport, symptom, and type of exposure	RT-PCR test results, no. (%)		
	Total	Positive	Negative
Total athletes*	**1,830 (100.0)**	**458 (100.0)**	**1,372 (100.0)**
**Sport played**			
Football	846 (46.2)	249 (54.4)	597 (43.5)
Track and field/Cross country	190 (10.4)	23 (5.0)	167 (12.2)
Soccer	121 (6.6)	24 (5.2)	97 (7.1)
Basketball	116 (6.3)	23 (5.0)	93 (6.8)
Volleyball	107 (5.9)	25 (5.5)	82 (6.0)
Swimming and diving	78 (4.3)	31 (6.8)	47 (3.4)
Baseball	72 (3.9)	8 (1.8)	64 (4.7)
Wrestling	60 (3.3)	18 (3.9)	42 (3.1)
Golf	46 (2.5)	7 (1.5)	39 (2.8)
Gymnastics	44 (2.4)	7 (1.5)	37 (2.7)
Softball	37 (2.0)	5 (1.1)	32 (2.3)
Lacrosse	26 (1.4)	13 (2.8)	13 (1.0)
Tennis	21 (1.2)	10 (2.2)	11 (0.8)
Rowing	19 (1.0)	5 (1.1)	14 (1.0)
Multiple sports^†^	6 (0.3)	1 (0.2)	5 (0.4)
Other^§^	41 (2.2)	9 (2.0)	32 (2.3)
**Symptoms consistent with COVID-19^¶^**			
No	1,444 (78.9)	137 (29.9)	1,307 (95.3)
Yes	386 (21.1)	321 (70.1)	65 (4.7)
**Type of exposure**			
Sports setting	232 (12.7)	54 (11.8)	178 (13.0)
Roommates	580 (31.7)	134 (29.3)	446 (32.5)
Social gatherings	745 (40.7)	195 (42.6)	550 (40.1)
Multiple exposure types**	64 (3.5)	16 (3.5)	48 (3.5)
Other^††^	61 (3.3)	20 (4.4)	41 (3.0)
Unspecified exposure	148 (8.1)	39 (8.5)	109 (7.9)

**Abbreviations:** COVID-19 = coronavirus disease 2019; RT-PCR = reverse transcription–polymerase chain reaction.

* Three colleges or universities provided data sets that only included athletes who had positive test results during quarantine. The athletes in these data sets (193) were not included in this analysis.

^†^ Six athletes indicated playing multiple sports; one of these athletes had a positive test result and participated in football and gymnastics; the others did not have positive test results and participated in football and wrestling (two), wrestling and track (one), golf and wrestling (one), and football and volleyball (one).

^§^ Sports with fewer than 10 athletes each (beach volleyball, cheer, dance, equestrian, rifle, skiing, and water polo) were included in the “other” category.

^¶^ Shortness of breath or difficulty breathing; cough or other respiratory symptoms; headache; chills; muscle aches; sore throat; congestion or runny nose; new loss of taste or smell; nausea, vomiting or diarrhea; pain, redness, swelling or rash on toes or fingers; new rash or other skin symptoms, temperature of ≥100.4°F (38°C).

** Sixty-four athletes reported more than one type of exposure.

^††^ Class, meetings, shared airplane, travel not related to sports, unsanctioned workouts, or work.

Three colleges and universities contributed data only on quarantined athletes who received positive test results during quarantine (193); after exclusion of 31 who did not meet inclusion criteria, 162 athletes remained. Therefore, a total of 620 athletes with positive SARS-CoV-2 test results during quarantine were included in a time-to-event analysis. Among 436 (73.4%) of these athletes with available exposure date, quarantine commenced a mean of 1.1 days after reported exposure (range = 0–11 days). Among these athletes, 302 (48.7%) reported symptoms before collection of the specimen that returned positive; the mean interval from symptom onset to positive specimen collection date was 1.1 days (range = 0–12 days). Among the 620 athletes with positive test results, the positive test results occurred by day 2 of quarantine for 303 (48. 9%) (Figure 1) and by day 5 for 453 (73.1%) with a mean of 3.8 days from quarantine start (range 0–14 days) until the positive test (Supplementary Figure, https://stacks.cdc.gov/view/cdc/99765). Among all SARS-CoV-2 RT-PCR tests administered, the positivity rate decreased over the quarantine period (Figure 2). The median interval between the start of quarantine and collection of a positive specimen was 2 days (range = 0–14 days). Among those whose test results remained negative at day 5, the estimated probability of having a positive test result was 26.9% (95% CI = 23.7–30.7) after day 5, 14.2% (95% CI = 11.7–17.2) after day 7, and 4.7% (95% CI = 3.3–6.7) after day 10. Among the 29 athletes who received positive test results during days 11–14, 26 (89.7%) had not been tested previously during their quarantine period.

**FIGURE 1:**
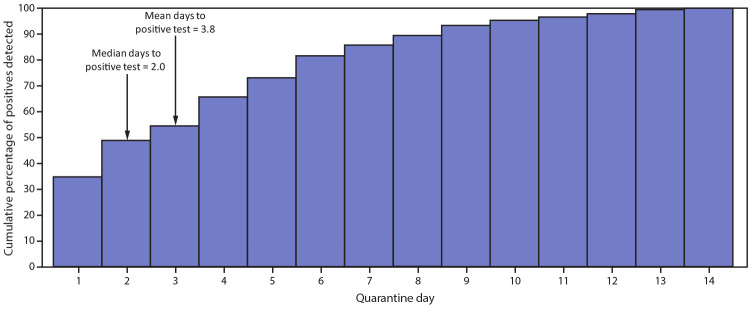
Cumulative percentage of SARS-CoV-2 reverse transcription–polymerase chain reaction positive test results among quarantined collegiate athletes who ever had a positive result, by day since start of quarantine — 17 states, June–October 2020

**FIGURE 2:**
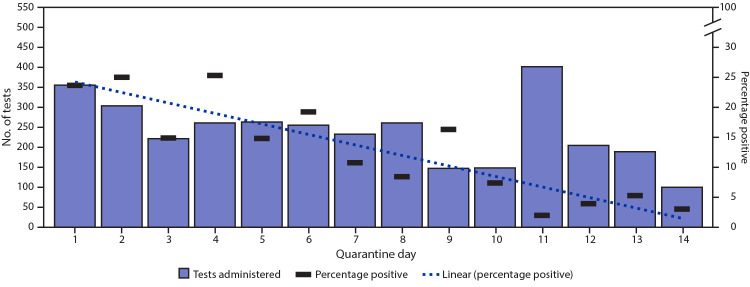
Number of SARS-CoV-2 reverse transcription–polymerase chain reaction tests performed and percentage positive* among quarantined collegiate athletes, by quarantine day — 17 states, June–October 2020 * With line of best fit for percentage positive over time.

## Discussion

A positive SARS-CoV-2 test result was received by one quarter of quarantined athletes during June–October 2020. Once an athlete entered quarantine, the probability of a positive test result among those who had no previous positive test result decreased from 27% after day 5 to <5% after day 10. New shortened quarantine options (after day 10 without testing or after day 7 with negative test result) were based on decreasing transmission risk over the duration of quarantine (*5*). Findings from this investigation support shortened quarantine options for collegiate athletes, given the low proportion of athletes who had positive test results after day 10.

Multiple, concurrent mitigation measures can effectively lower the risk for SARS-CoV-2 transmission (*7*). The majority of exposures among these athletes occurred at social gatherings and from roommates, indicating that the implementation of targeted mitigation measures helped minimize exposures associated with athletic activities. These findings underscore the need for adherence to mitigation measures (e.g., universal masking, physical distancing, and hand hygiene) at all times to reduce the risk for transmission of SARS-CoV-2 (*1*,*6*). In this cohort of collegiate athletes, fewer than one half who ultimately had a positive test result had symptoms consistent with COVID-19 before collection of the positive specimen. This finding is consistent with recent reports of asymptomatic screening programs in general university campus populations, where 51% of students with positive test results were asymptomatic (*8*). In this study, 86% of quarantined athletes who ever had positive test results did so by day 7, which is consistent with a reported median incubation period of 4.3–6.4 days (*9*,*10*).

The findings in this report are subject to at least five limitations. First, almost all athletes who had a positive specimen collected after quarantine day 10 had not been tested previously during quarantine. It is possible that those athletes might have had positive test results before day 10. Second, time-to-event analyses used quarantine start date rather than exposure date because more data were missing on exposure date than on quarantine start date; also, using exposure date was subject to validation errors. Therefore, using the quarantine start date rather than exposure date likely resulted in conservative estimates of the probability of receiving positive test results in quarantine. Third, adherence with quarantine was not assessed, and quarantined athletes possibly had additional exposures leading to infection during the 14-day quarantine. This might explain positive specimens collected after day 10 and could have overestimated the probability of a positive test result after day 10. Fourth, the data relied on self-reported exposure type. Although data were validated by case investigation and contact tracing efforts, 8.1% of athletes reported unspecified exposures, and 3.5% reported multiple exposures. Finally, this study was undertaken in a population of young, healthy athletes undergoing frequent testing. The findings might not be generalizable to other settings and populations.

Data from this report support CDC’s guidance on quarantine options shorter than 14 days, with the caveat that a small residual increased risk for transmission remains with a shortened quarantine period. Persons released from quarantine before 14 days should continue daily symptom monitoring, avoid close contact and wear masks when around others. Adherence to quarantine is a known challenge (*5*) and reducing the duration of quarantine might improve adherence at a population level. Transmission of SARS-CoV-2 can be reduced through a multipronged application of evidence-based mitigation strategies (*7*), including within large congregate and crowded settings found at colleges and universities. Adherence to mitigation measures during athletic and nonathletic activities is important to protect collegiate athletes from SARS-CoV-2, lessen disruptions in play because of quarantine and isolation protocols, and reduce transmission to others in the community.

**Summary**

**What is already known about this topic?**

Quarantine after SARS-CoV-2 exposure is critical to preventing transmission. A 14-day quarantine can prevent further transmission but might be challenging to maintain.

**What is added by this report?**

Among collegiate athletes exposed to COVID-19, one quarter had positive test results during quarantine. Among athletes who had not received a positive test result by day 5, the probability of testing positive decreased from 27% after day 5 to <5% after day 10.

**What are the implications for public health practice?**

Among young, healthy athletes, the probability of receiving positive test results after day 10 of quarantine is low. A shorter quarantine after COVID-19 exposure could increase adherence but still poses a small residual risk for transmission.

## Footnotes

* Close contact is defined as being <6 feet apart for >15 minutes with an infected person.

† 45 C.F.R. part 46, 21 C.F.R. part 56; 42 U.S.C. Sect. 241(d); 5 U.S.C. Sect. 552a; 44 U.S.C. Sect. 3501 et seq.
